# Understanding Barriers to Early Rehabilitation Following the Surgery for Fragility Hip Fractures: A Single-Centre Audit

**DOI:** 10.7759/cureus.71827

**Published:** 2024-10-19

**Authors:** Tarik Al-Dahan, Siddhartha Murhekar, Mohammed Abed, Kapil Shinde

**Affiliations:** 1 Trauma and Orthopaedics, East Kent Hospitals University NHS (National Health Service) Foundation Trust, Canterbury, GBR; 2 Neurosciences, Southampton General Hospital NHS (National Health Service) Foundation Trust, Southampton, GBR

**Keywords:** delerium, geriatric hip fracture, physiotherapy, post-operative physiotherapy rehabilitation, post-op haemoglobin drop

## Abstract

Introduction

Hip fractures are amongst the most common reasons for geriatric patients’ admission to the hospital; recovery of the hip fracture patient can be enhanced by an early rehabilitation program, which should start from the time of admission. This audit aims to study the hip fracture physiotherapy rehabilitation program at a local hospital to ensure compliance with the national guidelines and to identify any potential barriers to early mobilisation after a hip fracture operation.

Methods

The audit was approved by the trust's audit office. A retrospective study in the Queen Elizabeth The Queen Mother Hospital in Margate, Kent, UK, has been conducted including 113 patients admitted from April to August 2023 to our local hospital; the patients were screened for the inclusion and exclusion criteria, and data collection started regarding the admitting wards and their physiotherapy assessments including the time they were first assessed by a physiotherapist and the time of first mobilisation after the hip fracture operation and any identified barriers to early mobilisation.

Results

There were 113 patients who underwent hip fracture surgery, including 23 males and 90 females. The mean age of the participants was 82.7 years. Ninety-three percent (n=106/113) of the patients were seen by a physiotherapist on the day or the day following the surgery for their hip fracture. Amongst the admitted patients, 40% (n=46/113) experienced delayed mobilisation. The reason for the delay was post-operative delirium (n=15/46), low haemoglobin (n=12/46), pain (n=10/46), dementia (n=2/46) and no documented reason (n=7/46).

Conclusion

The study outcomes emphasise the importance of admitting hip fracture patients into specialised orthopaedic wards. The study identified post-operative delirium, low haemoglobin, pain and pre-existing dementia as factors that delay rehabilitation. Most of these factors are preventable or manageable, which can facilitate early rehabilitation and subsequently hospital discharge.

## Introduction

Hip fractures are amongst the most common reasons for geriatric patients’ admission to the hospital [1]. A large number of geriatric admissions put significant pressure on the admitting hospitals, highlighting the importance of early and rapid rehabilitation to facilitate patients' discharge from the hospital. After treatment of the hip fracture, the resultant post-operative pain and hospital environment may aggravate the muscle atrophy and weakening resulting in a cycle of further falls and increased risks of fractures [2].

Recovery of the hip fracture patient can be enhanced by an early rehabilitation program which should start from the time of admission; this may involve multiple teams like physiotherapy, occupational therapy, orthogeriatric, nutritional therapy and social care [2]. Studies have shown that only 40% of patients treated for hip fracture regained their pre-fracture mobility level, while 70% of treated patients regained activity levels adequate for basic daily living activities [3].

Early and realistic explanations about the course of treatment and rehabilitation to the patients and their families have been shown to improve outcomes as the patients retain a sense of self-control [4]; communication with the patients and their families is important as the rehabilitation programs are likely to extend for months following the hip fracture at the hospital and the community levels [5]. An early and effective rehabilitation training has been found to reduce mortality and restore the ability to perform activities of daily living earlier following the hip fracture [6]. Current research has identified an association between early physiotherapy and faster discharge from the hospital, and these findings may be irrespective of other factors such as dementia, delirium and pre-fracture mobility level [7,8].

This audit aims to study the hip fracture physiotherapy rehabilitation program in our local hospital in terms of compliance with the national guidelines; it also aims to identify any potential barriers and their effect to early mobilisation after hip fracture operation as identified by the physiotherapy team. These barriers include post-operative pain and delirium, low haemoglobin and pre-existing dementia. Our null hypothesis states that there is no difference in the physiotherapy services between the main orthopaedic ward and outlier wards.

## Materials and methods

The project was submitted to the trust’s audit office and gained its approval (ID: RN1166392). We agreed for the study design to be a retrospective study and obtained information about the admitted patients with fragility hip fractures from our local hip fracture database. The search strategy included using the (NHS number) as a patient identifier from the hip fracture database to find every patient’s records from our online system for patients’ records; we were then able to access the patients’ results, operative notes and the daily ward rounds, which included physiotherapy team assessment.

The total included patients in this study were 113 patients admitted to the Queen Elizabeth The Queen Mother Hospital in Margate, Kent, UK, from April to August 2023; for each of the included patients, we collected data about their age, gender, the type of operation they had for their hip fracture, the time they were mobilised and, if not, the reason for non-mobilisation. We also collected data about the duration of the physiotherapy and the adequacy of the documentation of the physiotherapy team aiming to compare to the national guidelines. We documented the wards (main orthopaedic ward and the outliers) to which those patients were admitted in order to discover any physiotherapy services differences in different wards at our local hospital.

This study included patients admitted with fragility hip fractures. The exclusion criteria were patients under palliative care and patients under end-of-life care. The authors reviewed the patients' notes and extracted the data for each patient into an Excel sheet using Microsoft Excel Software (Redmond, WA, United States) to build a basic database for the study, perform data analysis and create figures and tables. To test statistical significance, an independent T-test was used to calculate the P values using the SPSS software (Version 29.0.1.0; IBM Corp. in Armonk, NY, United States) for data analysis. The authors did not use a correction method for the P values due to the small sample size, predetermined comparisons and in order not to miss any possible significant association and reduction of the statistical power.

## Results

There were 113 patients: 23 males and 90 females, and all of them had an operation for a hip fracture, and the mean age of the participants was 82.7 years ranging from 58 to 103 years. Of the included patients, 85 patients were admitted to the main orthopaedic ward, while 28 patients were admitted into the outliers; Table 1 shows some patients' demographics.

**Table 1:** Patients demographics

Characteristics	Males	Females
Gender	23 (20%)	90 (80%)
Age		
50-59	-	1 (0.8%)
60-69	2 (1.7%)	6 (5.3%)
70-79	6 (5.3%)	22 (19.5%)
80-89	11 (9.7%)	40 (35.4%)
90 +	4 (3.5%)	21 (18.6%)
Type of operation		
Dynamic hip screw	2 (1.7%)	11 (9.7%)
Hip hemiarthroplasty	15 (13.3%)	44 (39%)
Intramedullary nail	6 (5.3%)	33 (29.2%)
Total hip replacement	-	2 (1.8%)
Ward admitted		
Main orthopaedic ward	13 (11.5%)	72 (63.7%)
Outliers	10 (8.8%)	18 (15.9%)

Out of all the patient records that were analysed, 93% (n=106/113) of the patients were seen by a physiotherapist on the day or the day following the surgery for their hip fracture, in terms of wards, this can be divided as 94% (n=80/85) of the patients in the main orthopaedic ward and 93% (n=21/28) in the outliers (t(32)=-2.1, P=0.3), with moderate effect size, d=-0.6 (95% CI: -1.06, -0.2).

Regarding the mobilisation, of the admitted patients, 40% (n=46/113) had delayed mobilisation; the reason for the delay was post-operative delirium 32.6% (n=15/46), low haemoglobin 26% (n=12/46), pain 21.7% (n=10/46), dementia 4.3% (n=2/46) and no documented reason 15.2% (n=7/46). There was a significant association between the presence of one or more of these factors post-operatively and the one-year mortality rate of 25.8% (n=16/62) t (62)=-1.9, P=0.05, Cohen’s d=-0.4, 95% CI: -0.8, 0.02. Post-operative delirium was the single most important factor associated with one-year mortality t (14)=-2.8, (P=0.01) Cohen’s d=-1, 95% CI: -1.5, -0.4.

A clear method of recording getting out of bed was found in 85% (n=97/113) of the mobilised patients. In terms of duration of the physiotherapy, 83% (n=94/113) of all the included patients had two hours of physiotherapy in the first week following their hip fracture surgery. Table 2 summarises the key results.

**Table 2:** Summary of the results

Aspect	Percentage	Sample size (n)
Patients seen by physiotherapy on the first day (main orthopaedic ward)	94%	80/85
Patients seen by physiotherapy on the first day (outlier)	93%	21/28
Delayed mobilisation following surgery	40%	46/113
Two hours of physiotherapy in first week	83%	94/113
Clear documentation of mobilisation	85%	97/113
Reasons for the delay in mobilisation		
Delirium	32.6%	15/46
Low haemoglobin	26%	12/46
Pain	21.7%	10/46
Dementia	4.3%	2/46
No documented reason	15.2%	7/46

## Discussion

This study addresses the physiotherapy services in a local hospital, particularly for the neck of femur fracture patients, and its adherence to the national guidelines; it also addresses some of the patient factors that alter the rehabilitation for hip fracture; this may highlight that rehabilitation can be improved with early intervention to address these factors which can improve the outcomes after hip fracture surgery and facilitate hospital discharge. The findings of our study suggest four possible causes of delayed rehabilitation after hip fracture surgery: post-operative delirium, dementia, low haemoglobin and post-operative pain. It is well known that some of the factors are difficult to manage. Still, others are reversible and can be addressed from admission to the hospital, such as correction of any pre-existing anaemia, management of anticoagulants and pain management. Our study also suggests that the presence of one or more of these factors was significantly associated with the one-year mortality after the hip fracture.

It was found that providing at least two hours of physiotherapy in the first week after admission for a hip fracture may lower the 30-day mortality rate by 8% and the 30-day re-admission rate by 6% [9]. Factors that may affect early mobilisation and engagement with the physiotherapy program may include confusion, hypotension, fatigue and pain [10,11]. The National Institute of Healthcare and Excellence (NICE) recommends mobilising patients with hip fractures on the day of or the day after the surgery [5]. In 2017, the Hip Fracture Sprint Audit carried out across England and Wales identified that only 68% of patients were out of their bed on the day of or the day following the surgery and 7% of the involved hospital sites achieved this for fewer than half of the patients; it has also identified that patients averaged two hours of physiotherapy in the first week after surgery, but 43% of patients missed a day of physiotherapy due to unavailability [12]. Therefore, The Chartered Society of Physiotherapy has developed standards for hip fracture rehabilitation to minimise the variation in outcomes after hip fracture surgery [13].

Post-operative delirium was the most common factor associated with delaying early post-operative mobilisation and physiotherapy in our cohort; this addresses the importance of a multidisciplinary approach to managing delirium and its predisposing factors. In our study, delirium was the most critical single factor associated with one-year mortality; this finding is similar to a study that identified post-operative delirium as an important factor in mortality and a major contributor to post-operative morbidity [14]. Rehabilitation after a hip fracture is complex; many of these patients often have dementia and cognitive impairment. As a result, they require additional care and have greater risks of complications [15]. Dementia patients often deteriorate further in the hospital setting, and they have difficulty in expressing their pain, nausea and dizziness, predisposing them to delirium with its detrimental outcomes, making engaging with physiotherapy more difficult [2].

In the present study, symptomatic anaemia, post-operative hypotension and dizziness were a major cause of delayed mobility in about a third of non-mobilised patients in the early post-operative period; it is well known that anaemia greatly decreases the functional capacity of post-operative patients. A large study has found that a high post-operative haemoglobin level in hip fracture patients was an independent factor associated with greater walking distance [16]. It is believed that the blood loss in hip fracture surgery is more than what is calculated intraoperatively, with estimates of additional hidden loss between 500 ml to 1,500 ml depending on many factors like the type of operation and the use of any anticoagulants [17].

Post-operative pain is one of the most common post-operative consequences of hip fracture surgery and could be aggravated by movement and changing posture after the surgery [18]. In the present study, about 22% of non-mobilised patients were due to pain. It is often challenging to prescribe pain medications and opioids to levels adequate for effective pain control in elderly patients with polypharmacy, which necessitates additional measures like epidural blocks and fascia iliaca blocks, which are implemented in our hospital. Other factors affecting patients outcome include adequate staffing of physiotherapy teams and availability during weekend admissions and out of hours. Our findings suggest that patients admission to specialised orthopaedic wards provides better post-operative care. Effective documentation is a critical aspect in patients management as it influences the understanding of barriers to compliance with the mobilisation strategies; using an online documentation platform provides easy navigation through patients' records ensuring easy-to-follow management plans and patients' clinical conditions eliminating the human factor error in interpreting the handwriting and spelling errors.

The retrospective nature of our study posed inherent limitations, particularly in fully identifying the confounding factors such as motivation and the time of admission (weekend) affecting early mobility as we depend on previously entered registers for the data collection. This highlights the need for future prospective studies to address confounding factors delaying rehabilitation following hip fracture surgery. The strengths of this study include the cohort of patients studied from an orthopaedic department in a hospital that treats hip fractures on a daily basis with much-experienced surgeons, physiotherapists and nursing staff. Digitalising patient records avoids the bias of misinterpreting the data due to poor handwriting.

## Conclusions

The study outcomes emphasise the importance of admitting hip fracture patients into specialised orthopaedic wards with experienced physiotherapists and nursing staff with rehabilitation after a hip fracture as those patients frequently have pre-existing co-morbidities and need special attention to their post-operative rehabilitation. The study identified post-operative delirium, low haemoglobin, pain and pre-existing dementia as factors that delay rehabilitation; most of these factors are preventable or manageable, which can facilitate early rehabilitation and subsequent hospital discharge.
